# Reply to Benito et al.: Problems in the Cretaceous evolution of the avian palatobasal joint

**DOI:** 10.1073/pnas.2520865123

**Published:** 2026-01-02

**Authors:** Alec T. Wilken, Kaleb C. Sellers, Julian L. Davis, Lawrence M. Witmer, Casey M. Holliday

**Affiliations:** ^a^Department of Organismal Biology and Anatomy, University of Chicago, Chicago, IL 60637; ^b^Department of Oral Biology, University of Illinois, Chicago, IL 60612; ^c^Department of Engineering, University of Southern Indiana, Evansville, IN 47712; ^d^Department of Biomedical Sciences, Heritage College of Osteopathic Medicine, Ohio Center for Ecology and Evolutionary Studies, Ohio University, Athens, OH 45701; ^e^Department of Pathology and Anatomical Sciences, University of Missouri, Columbia, MO 65212

Based on new arguments (1), we agree the *Janavis* element is a pterygoid (1, 2), but question its taxonomic affinities among Mesozoic birds. Given the Maastricht Formation’s disassociated mix of plant, invertebrate, fish, and other avian fossils (2, 3), we urge caution presuming all the material from the nodule is of the same individual or even taxon. We lack a pterygoid from sympatric and later-diverging basal galloanserans like *Asteriornis* (3). We expect these taxa to have a grade of pterygoid more akin to that found in *Janavis* than earlier diverging ornithuromorphs like *Ichthyornis* [see the pterygoid of *Hesperornis* (4)]. If early galloanserans and ichthyornithids were both preserved in the same formation, it is possible material was mixed. The description of *Janavis* highlights the pterygoid’s affinities with galloanseran pterygoids (1, 2), yet the braincase anatomy of *Ichthyornis* (putatively also affined with Janavis) does not match the same grade or morphology.

**Fig. 1.**
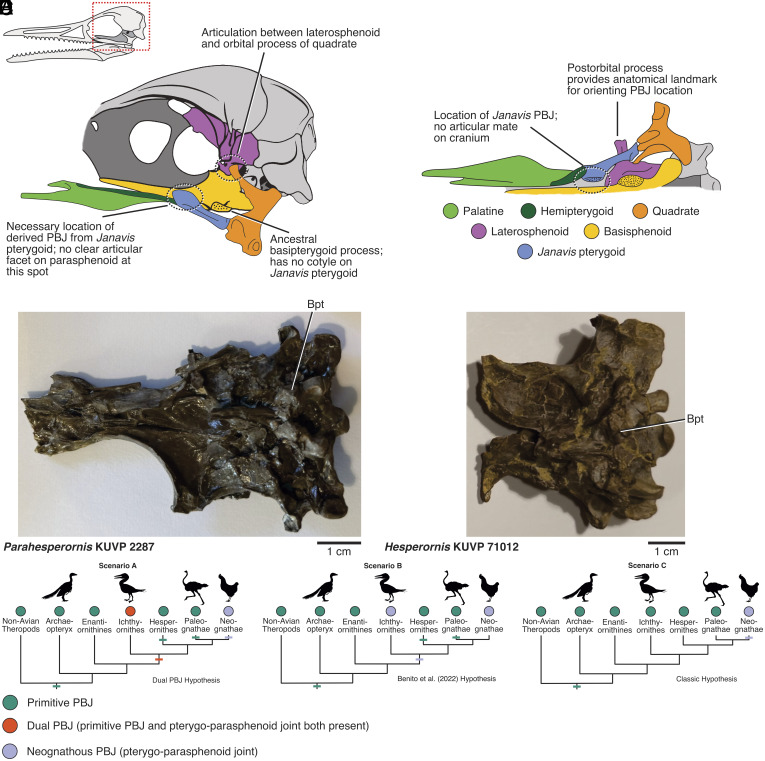
Braincase–palate connections in the *Ichthyornis/Janavis* chimera and their implications for avian palate evolution. (*A*) Left lateral view of the *Ichthyornis/Janavis* chimera, modified from ref. 6 and traced from ref. 2. (*B*) Ventral view of the *Ichthyornis/Janavis* chimera, traced from ref. 2, figure 2 and *SI Appendix*, Extended Data Fig. 10. (*C*), Ventral view of the braincase of *Parahesperornis*. (*D*) Ventral view of the braincase of *Hesperornis.* (*E*) Hypothesized scenarios of PBJ evolution. We encourage the reader to view ref. 6. Figure 4*A* for a photograph of the braincase of *Ichthyornis*. The only probable mate for the articular facet on the *Janavis* pterygoid is a region of flattened (crushed) parasphenoid. Evidence for the primitive PBJ can be found in all dinosaur lineages except neognath birds, supporting Scenario C.

Accepting the *Janavis* element as an ichthyornithine pterygoid introduces anatomical incongruencies (Fig. 1). Benito et al. (1) do not dispute the identification of a basipterygoid process on the braincase of *Ichthyornis* (5, 6) found in the primitive position shared with all non-neognath dinosaurs, including paleognaths and *Hesperornis* (4, 7, 8). However, the *Janavis* pterygoid only has a rostral parasphenoid articular contact (2), like neognath birds (7–9), with no obvious mate on the parasphenoid of *Ichthyornis* (Fig. 1; and 6). The *Janavis/Ichthyornis* chimera (1, 2) fails the test of conjunction because there cannot be two homologous palatobasal joints (PBJs) in the same taxon, or it necessitates two coexisting PBJs (a dual PBJ phenotype) in ichthyornithids. Thus, there is either considerable missing palatal diversity between these two sister lineages or ongoing questions about the osteology of these fossils (Fig. 1).

**Fig. 2.**
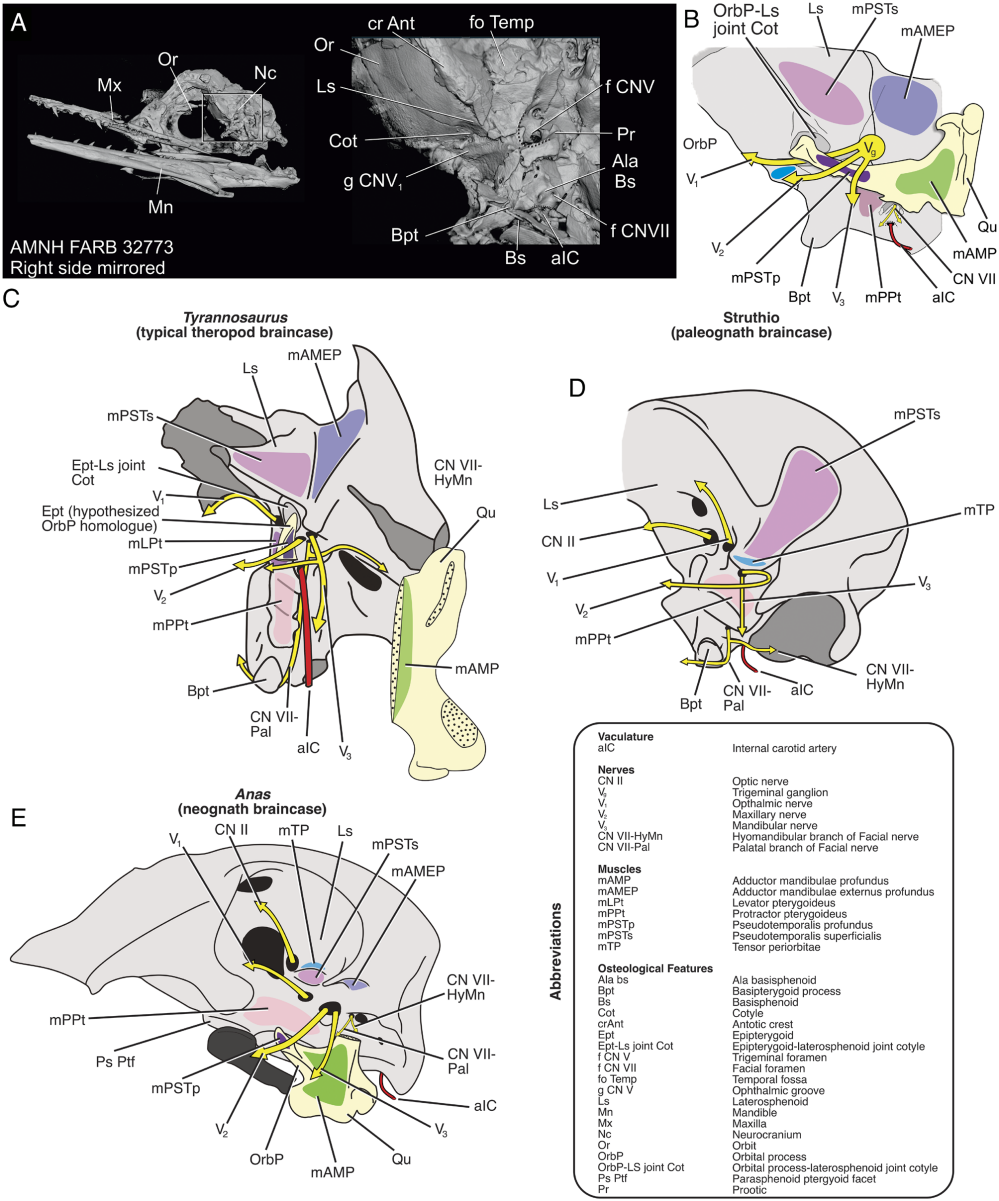
Comparative anatomical topology of the braincase in *Tyrannosaurus, Ichthyornis, Struthio,* and *Anas*. (*A*) CT volume reconstruction of the braincase of *Ichthyornis.* (*B*) Soft tissue reconstruction of the braincase of *Ichthyornis.* (*C*) Soft tissue reconstruction of the braincase of *Tyrannosaurus.* (*D*) Soft tissue anatomy of the braincase of *Struthio.* (*E*) Soft tissue reconstruction of the braincase of *Anas*. Given the identified structures on the braincase of *Ichthyornis,* a cotyle for the orbital process, homologous with the cotyle for the epipterygoid, is the best anatomically supported candidate for the triangular fossa found under the laterosphenoid in *Ichthyornis.* The spatial relationships of these features are deeply conserved, meaning we would not expect to find the basispterygoid process in a different location in sister lineages like *Janavis* and *Ichthyornis.* The features of the braincase do not agree with a neognath-like PBJ for ichthyornithids, as suggested by the *Janavis* pterygoid.

An articulation between the orbital process of the quadrate and laterosphenoid is anatomically supported (6; Fig. 2). Few fossae mark the boundary between the temporal and orbital regions except for the epipterygoid joint, ala basiphenoid, and key muscle scars (7–9). Given the deeply conserved constellation of identifiable structures in *Ichthyornis*, the small fossa just ventral to the postorbital process is most congruently identified as the epipterygoid cotyle (i.e., orbital process cotyle) (Fig. 2; and 7–9) A similar cotyle is present in *Hesperornis* (4). More evidence for this joint can be found in experimentally paralyzed chick embryos, which retain an orbital process-laterosphenoid articulation (10), developmentally recapitulating the hypothesized articulation in *Ichthyornis* and the known epipterygoid articulation in nonavian theropods (8). The available anatomical and phylogenetic data do not support the presence of a neognath pterygoid in ichthyornithids.

Crucially, *Ichthyornis* lacks protractor muscles positioned to actuate kinetic excursions required for *powered* prokinesis (6, 7). Stem birds like *Ichthyornis* lack the necessary set of musculoskeletal characters for powered prokinesis and paleognaths use their palate and protractor muscles to actively resist kinesis, despite having a permissive linkage system (6, 11). Even a generously revised linkage analysis of the *Ichthyornis* cranium (1) that assumes untested convergence with the flexible faces of various extant neognaths does not alter the conclusion that *powered* prokinesis is an autapomorphy of neognath birds (6).
